# The impact of trunk impairment on performance of wheelchair activities with a focus on wheelchair court sports: a systematic review

**DOI:** 10.1186/s40798-015-0013-0

**Published:** 2015-05-07

**Authors:** Viola C Altmann, Anne L Hart, Yves C Vanlandewijck, Jacques van Limbeek, Miranda L van Hooff

**Affiliations:** 1 Rehabilitation Centre, Sint Maartenskliniek, P.O. Box 9011, 6500 GM Nijmegen, The Netherlands; 2 Department of Physical Therapy and Athlete Training, Northern Arizona University, Flagstaff, Arizona USA; 3 Department of Rehabilitation sciences, Katholieke Universiteit Leuven, Leuven, Belgium; 4 Achmea Health Insurance Company, Amersfoort, The Netherlands; 5 Sint Maartenskliniek, Research, Nijmegen, The Netherlands

## Abstract

**Background:**

Trunk impairment seems to impact significantly on performance in wheelchair court sports, but evidence to support this impression has never been systematically assessed. The objective of this study is to systematically review, describe and synthesise the literature investigating the impact of trunk impairment on wheelchair activities in court sports.

**Methods:**

This systematic review was performed according to the consensus statement for the meta-analysis of observational studies in epidemiology (MOOSE). The search strategy for original articles comprised Medline (1950- November 2014), Cinahl (1981-November 2014), and Embase (1980- November 2014), using the search terms: trunk, trunk muscles, postural balance, posture and wheelchair.

Eligibility criteria for further review were 1) participants included experienced wheelchair users, 2) comparisons were made between a) participants with different levels of trunk impairment or b) between able bodied participants and participants with trunk impairment, or c) between participants with trunk impairment with and without compensatory equipment, and 3) outcome measures were quantitative data on wheelchair activities. For methodological quality assessment, the STROBE (Strengthening the reporting of observational studies in epidemiology) checklist was used.

**Results:**

After assessment of 358 potentially relevant studies for the eligibility criteria, 25 studies were appropriate for methodological assessment. Twelve articles fulfilled the predetermined minimum of 15 reported items on the 22-item STROBE checklist. These studies were limited to observational studies with small populations. All but one study were restricted to patients with spinal cord injury (SCI).

**Conclusions:**

Limited evidence was found about the impact of trunk impairment on wheelchair activities. Reach to the front and multidirectional reach was further in able bodied persons than in persons with SCI. In a perturbation that equals deceleration in wheelchair court sports, able bodied persons maintain balance, whereas persons with SCI lose balance. No evidence was found to support a difference in acceleration between persons with partial trunk muscle strength and persons with full trunk muscle strength. For future research, there is a need for a test that includes all types of trunk impairment and identification of activities that determine performance in wheelchair court sports. Furthermore, populations of athletes with all trunk impairment types should be included.

## Background

Classification is a process in which a single group of entities are ordered into a number of smaller groups on the basis of observable properties. In health and health-related domains, this basis for classification is impairment in body structure and function [1,2]. In paralympic sports, classification of impairment in athletes is vital to minimise the impact of impairment on the outcome of competition. Without classification, paralympic sport competition could potentially have a one-sided and predictable outcome, in which athletes with the least impairment would have the best chance to win. Classification systems have been developed and applied since the founding of the paralympic movement [3]. All paralympic classification systems evolved from a medical assessment model, developed based on expert opinion by classifiers. Typically, these individuals are volunteers with medical or health-related professional training and/or sport-specific expertise. In 2007, the International Paralympic Committee (IPC) published the *IPC Classification Code and International Standards* to provide a structure for classification principles for all paralympic sports [4]. The IPC Classification Code states that classification in the paralympic movement should be based on eligible impairment types per sport and the effect of these impairments on sport-specific activity, irrespective of the health condition causing the impairment. An important purpose of the *Classification Code* is to charge international sports federations with the development of evidence-based classification systems through research. An evidence-based classification system is a requirement for each sport federation to maintain compliance with the code and, consequently, preserve membership in the paralympic movement. The IPC position stand, published in 2009, defined evidence-based classification and provided guidelines on how an evidence-based classification system may be achieved [2].

Similar to other paralympic sports, the classification system in wheelchair rugby is based on expert opinion. Wheelchair rugby is a sport originally developed for athletes with tetraplegia due to complete spinal cord injury (SCI). The fast-paced sport has grown to include athletes with all biomechanical impairment types as defined by IPC: impaired muscle strength, impaired passive range of motion, limb deficiency, leg length difference, short stature, hypertonia, ataxia and athetosis [2,5]. An increasing number of athletes with eligible impairment types resulting from health conditions such as incomplete SCI, congenital or acquired limb deficiencies and cerebral palsy entered the sport. These athletes differ in the type and degree of impairment from those of the pioneers in the sport. These athletes have partial to no impairment in muscle strength, coordination and range of motion of the trunk. By observing athletes with and without trunk impairment, classifiers, athletes and stakeholders in wheelchair rugby perceived a significant impact of trunk impairment on performance in wheelchair activities. Trunk impairment did not seem adequately considered in the classification system [6].

To incorporate the principles of evidence-based practice to improve classification of trunk impairment, an exploration of the available literature was deemed necessary. Although classification systems for other paralympic sports, including wheelchair basketball, Nordic skiing and athletics, examine trunk impairment, these systems are largely based on expert opinion, similar to the classification system for wheelchair rugby [7-9]. Previous research indicates trunk muscle strength, trunk coordination and trunk range of movement determine: a) trunk position which impacts on force application on the hand rims, b) trunk stability which decreases paradoxical movements, making arm movements and force application more effective and c) trunk movement which determines the range of the push rim that can be used in each push stroke. Therefore, trunk impairment may impact significantly on wheelchair propulsion, especially in high resistance wheeling such as accelerating from standstill [10,11]. However, some significant activities determining wheelchair sport performance were not assessed (e.g. reach, turning and maintaining balance after perturbation). Furthermore, the impact of impairment on performance in wheelchair sports can be modified by equipment [12]. Athletes in wheelchair court sports always perform using equipment. The minimum equipment is a sports wheelchair that is usually custom made. In addition, many athletes use belts and straps to improve force generation. Athletes who have trunk impairment also use straps to increase stability. The use of equipment is not considered in determining the sports class according to the IPC classification code [4]. Therefore, to support evidence-based classification, the impact of equipment should be treated as a covariate.

The goal of the present study is to systematically review, describe and synthesise literature investigating the impact of impairment in muscle strength, coordination and range of movement of the trunk on performance in wheelchair activities, with a focus on wheelchair court sports. The primary question was proposed in wheelchair rugby; however, because wheelchair rugby has many commonalities with other court sports [12-14], the scope of this study includes all wheelchair court sports (including wheelchair rugby, basketball and tennis). There is no evidence-based set of tests that determines the performance in wheelchair activities in general or in any wheelchair sport [15]. Therefore, the existing literature about wheelchair activities that determine performance in wheelchair court sports was analysed [12-14,16-18]. This review resulted in the following activities: maintaining balance after perturbation, reach, propulsion, acceleration, change of direction and specific for wheelchair basketball and wheelchair rugby, tilting the chair. These activities matched those previously used in earlier research to assess the performance in wheelchair rugby [19-21]. The impact of equipment on performance in the presence of trunk impairment was dealt with as a covariate.

## Methods

This systematic review was conducted and reported according to the specifications referred to in the consensus statement for the meta-analysis of observational studies in epidemiology (MOOSE) [22]. Two independent researchers, VA and AH, performed the search for articles. Both are experienced wheelchair rugby classifiers; VA is a medical doctor and AH is a physiotherapist, PhD.

### Data sources

The search strategy for original articles was comprised of Medline (1950 to November 2014), Cinahl (1981 to November 2014) and Embase (1980 to November 2014). The following three groups of search terms were used: trunk/trunk muscles (replaced by torso and abdominal muscles as the closest matching MeSh terms in Medline), postural balance/posture and wheelchair. The terms within each group were concatenated by the Boolean OR and were performed independently of one another before each group was concatenated by the Boolean AND with the relevant health conditions of athletes playing wheelchair rugby: spinal cord injury, poliomyelitis, neuromuscular disease, cerebral palsy and amputation.

The search was extended using the option “related articles” in Medline and a manual search of all references of the included articles. First, the titles of the “related articles” and the references were screened. Only if the title indicated potential relevance, the abstract was further assessed and added to the numbers of identified records. To assess if any relevant unpublished research was available, one of the authors (VA) screened all abstracts over the past 8 years of several conferences, which were relevant in relation to the objectives of the review: the IPC Vista conferences in 2006, 2011 and 2013, the fourth and the fifth international state-of-the-art congress Rehabilitation: Mobility, Exercise and Sports 2009 and 2014 and the 2012 International Convention on Science, Education & Medicine in Sport. For any abstracts that matched the inclusion criteria, author VA contacted the authors to obtain unpublished manuscripts.

### Study selection

Studies were selected for further review if the following criteria met clinical relevance: 1) participants included experienced wheelchair users; 2) comparisons were made a) between participants with different levels of trunk impairment or b) between able bodied persons and persons with trunk impairment or c) between persons with trunk impairment with and without compensation by equipment and 3) if the outcome measures were wheelchair activities, presented in quantitative data. Furthermore, articles written in English were selected as well as German, French or Dutch, if the abstract was in English.

Studies were excluded: 1) if only able-bodied persons participated, 2) if no comparison of trunk impairment or compensation for trunk impairment was made, 3) if no definition of trunk impairment was reported or 4) also excluded were articles containing only qualitative data, expert opinions or case reports.

To identify potentially relevant articles, two reviewers (VA and AH) independently screened titles and abstracts. If one reviewer found an article, both reviewers screened for the inclusion criteria. If the inclusion criteria were met, the full text of the potential article was assessed. In case of disagreement between the reviewers about the inclusion criteria, a consensus procedure was used with four authors of the review (VA, AH, JvL and MvH). This consensus procedure consisted of an open discussion and a vote, if no consensus was reached after discussion. In case of a tie vote, the study would be included. Articles found more than once using the search strategies (doubles) were only included once.

### Quality assessment

Two reviewers (VA and AH) independently assessed the methodological quality of each study using the Strengthening The Reporting of Observational Studies in Epidemiology (STROBE) checklist for reports of observational studies [23]. The STROBE checklist consists of 22 items. Each item was scored as present (‘1’) or absent (‘0’). If an item contained sub items, the concerning item was scored present if at least 50% of the sub items were scored present. The STROBE recommendations do not provide a guideline or threshold for including meaningful studies in a systematic review. However, in a study performed on the methodological quality of observational studies published in high-quality journals, these authors found on average 69% of the STROBE items were reported [24]. Consistent with this study and with others using quantitative cut-off scores for observational studies, we decided that a minimum of 15 reported items (69%) indicated “good quality”, whereas 14 reported items or less indicated “moderate to low quality” [25].

When disagreement existed on any item of the STROBE checklist, the same consensus procedure applied for inclusion criteria was used with four authors of the review (VA, AH, JvL and MvH).

## Results

### Search results

**Figure 1:**
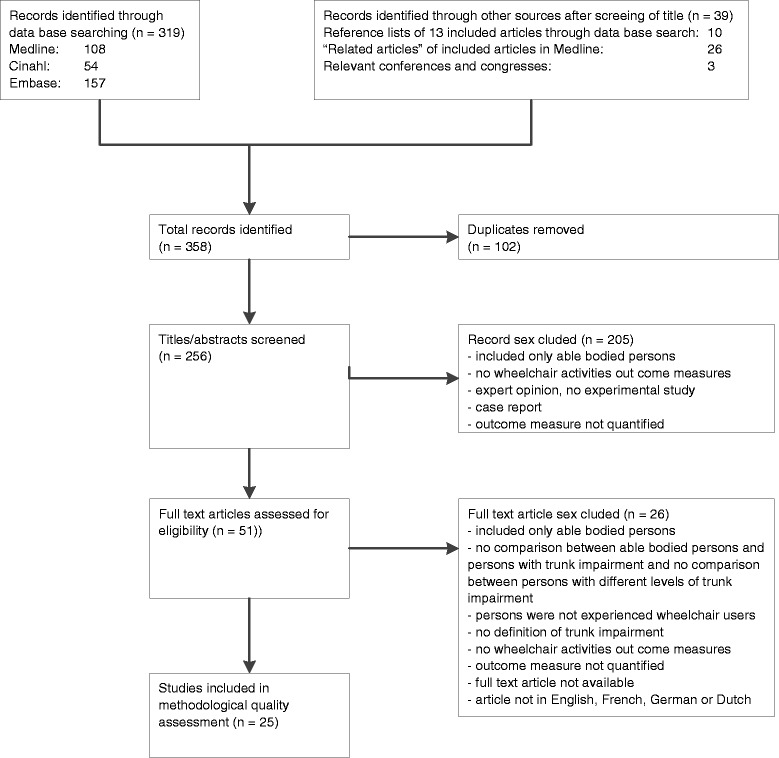
**Flow diagram of the literature search.**

Figure 1 shows the number of articles found following each step of the search strategy.

After database searching, the researchers found 319 studies, which were potentially relevant. After assessment for the eligibility criteria based on screening of titles and the abstracts and if indicated, assessment of the full article, the researchers reached consensus that 13 articles were eligible for methodological quality assessment. Most articles were excluded because the population did not consist of experienced wheelchair users, there was no definition of trunk impairment or the outcome measures did not include wheelchair activities. One article was excluded based on language. All references of the 13 articles that were eligible after the database searching were searched manually. Furthermore, those articles in Medline with the option “related articles” were screened. The 36 potentially relevant studies found by this manual search were also assessed for eligibility. After cross checking for duplicates with the 13 articles that were already eligible, 12 studies were added, so a total of 25 studies were eligible for methodological assessment.

The search for unpublished manuscripts resulted in three relevant abstracts: one abstract in which the reach in a seated position of wheelchair slalom athletes with cerebral palsy was compared to the reach of persons without any health condition [26], one abstract about static and dynamic sitting balance in wheelchair rugby and wheelchair basketball athletes [27] and one abstract about the relation to trunk muscle strength and sprinting performance [28]. Full text manuscripts were not available; therefore, these studies were excluded.

### Methodological quality

The results of the quality assessment of the 25 articles, eligible based on the inclusion criteria, are shown in Table 1.

**Table 1:** **Participants, interventions, comparison and outcome measures of all studies and quality score STROBE checklist**

**Reference no.**	**Study**	**Participants**	**Intervention**	**Comparison**	**Outcome measure**	**Total score STROBE**	**Methodological quality**
[49]	Bernard et al.	Six SCI T4-8 (high), six SCI T11-L5 (low) and six AB	Perturbation on moving platform for wheelchair	High SCI, low SCI, AB 4 oscillation levels	Damping factor of head	4	Moderate-poor
[29]	Boswell Ruys et al.	30 SCI C5-L2 AIS-D, 14 high level and 16 low level	Upper body sway = sit still unsupported for 30 s Maximal balance range forwards and backwards Coordinated stability = follow track with pencil by adjusting trunk position in bending and rotating.	New (<1 year post injury) vs. old (>1 year post injury High level = C6-T7 AIS A vs. low level = T8-L2 AIS A or incomplete AIS B-D with abdominal innervation	Reliability and validity of the tests	15	High
			Alternating reach test = tapping table eight times fast with and without arm support Seated reach distance lateral left and right Upper body dressing				
[30]	Chen et al.	Eight SCI T1-6 (high thoracic) 22 SCI T7-12 (low thoracic)	Sitting stability static (unsupported 30 s) and dynamic (30 s maximum leaning to four sides) Time needed for functional activities: upper body dressing, lower body dressing and transfer.	Low versus high thoracic SCI Trunk strength (hand held dynamometry), lesion level and trunk length in relation to sitting balance.	CoP displacement static and dynamic (sum score for all directions) Time to complete functional tasks	16	High
				Sitting balance in relation to functional tasks.			
[31]	Curtis et al.	Seven athletes with spinal cord injury; four in IWBF class 1 and three in IWBF class 2, nine AB	Reaching in sagittal and transverse plane	IWBF class 1 and 2, AB Belt at thigh and trunk versus no belt	Reaching distance in sagittal and transverse plane	16	High
[50]	De Abreu et al.	11 SCI T2-L2, AIS A-C, six AB	Reaching in anterior-posterior direction seated on different cushion types	AB versus SCI Different cushion types	Maximum reach Anterior displacement of the trunk Reaching time for 50%, 75% and 90% and maximum reach	13	Moderate-poor
[32]	Gauthier et al.	15 SCI: 9 “Abdo” (levelT7 or lower or active contraction abdominals to generate trunk flexion) 6 “No Abdo” (level higher than T7, no active contraction abdominals to generate trunk flexion) 15 AB	Move CoP to eight directions with 45^°^ interval	AB vs SCI “Abdo” versus SCI “No Abdo”	OSI (Overall Stability Index) DSI (Direction Specific Index of Stability)	17	High
[51]	Harel et al.	Seven SCI T1-T11 AIS A-B Seven AB	Static balance with eyes open (EO) and eyes closed (EC) Dynamic balance: leaning in multiple directions	AB versus SCI	Sitting items Berg Balance Scale Modified functional reach test	14	Moderate-poor
					Seated posturography: postural sway EO and EC Maximum excursion centre of gravity (CoG) and directional control		
[33]	Janssen-Potten et al.	Ten SCI T2-8 (high) Ten SCI T9-12 (low) Ten AB	Bimanual forward reaching task 15%, 30%, 75% and 90% of maximum	Standard chair (S) 7° tilt (7 T) 12° tilt (12 T) 22° recline (22R)	Maximum reaching distance CoP displacement	14	Moderate-poor
					EMG erector spinae T3, erector spinae T9, erector spinae L3, latissimus dorsi, trapezius pars ascendens, pectoralis major, serratus anterior, oblique abdominal muscles Reaction time and movement time		
[33]	Janssen-Potten et al.	Ten SCI T2-8 (high)	Bimanual forward reaching task 90% of maximum	High and low SCI	CoP displacement	15	High
		Ten SCI T9-12 (low)		Standard chair and 10° forward inclination	EMG erector spinae T3, erector spinae T9, erector spinae L3, latissimus dorsi, trapezius pars ascendens, pectoralis major, serratus anterior, oblique abdominal muscles Kinematics pelvis (tilt)		
		Ten AB					
[34]	Janssen-Potten et al.	Ten SCI T9-12 (low T) Ten SCI L1-5 (L) Ten AB	Bimanual forward reaching task 90% of maximum	Low T, L and AB With and without rigid footrest	Maximum displacement CoP (CoP max) Movement time	15	High
					EMG erector spinae T9, erector spinae L3, rectus abdominis, oblique abdominals, gluteus max, tensor fascia lata, rectus femoris, vastus lateralis, biceps femoris, semitendinosus, tibialis anterior, gastrocnemius medialis		
[35]	Kamper et al.	Four SCI C5-7 (tetraplegia) Four SCI T2-9 (paraplegia) Five AB	Tilting movement in frontal plane on servo controlled platform in standard WC	Tetraplegia-paraplegia-AB And tilting movements with high and low amplitude and acceleration	Balance loss FLCP = fraction of limit of CoP movement Velocity of CoP displacement Kinematic data Kinetic data (torque of pelvis, lower trunk and upper trunk)	17	High
[36]	Kamper et al.	Four SCI C5-7 (tetraplegia) Four SCI T2-9 (paraplegia) Five AB	Tilting movement in sagittal plane on servo controlled platform in standard WC	Tetraplegia-paraplegia-AB And tilting movements with high and low amplitude and acceleration	Balance loss FLCP = fraction of limit of CoP movement Kinematic data	17	High
[52]	Kerk et al.	Six SCI T3-6, absent abdominal muscles	Sub maximal and maximal exercise test on wheelchair roller in own WC	With and without elastic abdominal binder	Pushing stroke parameters, physiologic parameters, trunk movement	12	Moderate-poor
[37]	Potten et al.	Ten SCI T2-8 (high) Ten SCI T9-12 (low) Ten AB	Bimanual forward reaching task 15%, 30%, 75% and 90% maximum	High SCI, low SCI and AB	CoP displacement EMG serratus anterior, m. pectoralis major, latissimus dorsi, erector spinae T3, trapezius pars ascendens	16	High
[53]	Requejo et al.	Five SCI T4 and higher (high), five SCI T12 and lower (low)	Three pushing speeds (0.85, 1.03 and 1.21 m/s) and one self-selected speed, administering frequent small bumps	Low and high SCI, wheelchair with rear suspension (three types), and without rear suspension	Self-selected speed Vertical seat reaction force Head acceleration	10	Moderate-poor
[46]	Schantz et al.	Four SCI low thoracic, three SCI cervical	Comfortable and maximum pushing speed, maximum acceleration on gymnasium wooden floor	SCI low thoracic and SCI cervical	Maximum velocity and acceleration, EMG activity of arm muscles, trunk position and movement Reaction time Movement time EMG activity latissimus dorsi and trapezius pars ascendens	8	Moderate-poor
[54]	Seelen et al.	15 SCI T4-T12 15 AB	Reaching forward 30%, 60% and 90% of maximum after warning signal with and without cue	SCI and AB		11	Moderate-poor
[55]	Seelen et al.	15 SCI T2-8 (high) 15 SCI T9-12 (low)	Bimanual forward reaching task 15%, 30%, 75% and 90% of maximum	High and low SCI in experiment 1; none (only AB) in 2	CoP displacement EMG erector spinae T3, erector spinae T9, erector spinae L3, latissimus dorsi, trapezius pars ascendens, pectoralis major, serratus anterior, oblique abdominal muscles	14	Moderate-poor
		15 AB					
					Antero-posterior force component		
[56]	Seelen et al.	Five SCI T4-T8 (high), seven SCI T9-T12 (low)	Releasing push button on lap Reaching forward bilaterally	15%, 30%, 75% and 90% of maximum reach high and low SCI duration since SCI	Reaction time Movement time	13	Moderate-poor
[47]	Seelen et al.	Five SCI T4-8 (high) Seven SCI T9-12 (low)	Bimanual forward reaching task 15%, 30%, 75% and 90% of maximum	High and low SCI, different points in time Braced vs. non braced (post hoc)	CoP displacement EMG erector spinae T3, erector spinae T9, erector spinae L3, latissimus dorsi, trapezius pars ascendens, pectoralis major, serratus anterior, oblique abdominal muscles	13	moderate-poor
[38]	Seelen et al.	15 SCI T2-8 (high) 15 SCI T9-12 (low) 15 AB	Bimanual forward reaching task 15%, 30%, 75% and 90% of maximum	High and low SCI	Reaction time, movement time CoP displacement	15	High
[44]	Serra-Anno et al.	24 SCI higher than T9 (high); T9 and lower (low) 24 AB	Static sitting balance (ST) with eyes open (EO) and eyes closed (EC) Dynamic sitting balance (SLT)	High SCI, low SCI, AB	ST: signal amplitude, range, frequency spectrum in anterior-posterior and medial-lateral directions. SLT: maximum CoP displacement, efficient CoP displacement and normalised total excursion of CoP	13	Moderate-poor
[57]	Shin et al.	Seven SCI T10 and higher (high) 11 SCI T10-L1 (low) 18 AB	Functional reach test: reaching as far as possible with dominant hand	High SCI, low SCI, AB	Functional reach, velocity and CoP trajectory, functional boundary	13	Moderate-poor
[39]	Triolo et al.	Eight SCI low (T5-10) and high (C6-7) with implanted neuroprosthesis.	Seated bimanual reach: 30 in. = desktop and 48 in. = high shelf, loaded (20% of unilateral shoulder flexion strength) lifting a light or an heavier object	High and low SCI Stimulation on and off, Strong and weak based on volitional trunk extension strength with stimulation	Unsupported bimanual reaching distance with and without stimulation.	17	High
[40]	Vanlandewijck et al.	13 track athletes, three female no full trunk function, four male no full trunk function, six male full trunk function. Diagnosis: SCI, spina bifida, arthrogryposis, amputation	Maximum acceleration track and treadmill with 4× resistance	Male athletes full trunk function versus male athletes no full trunk function	Distance on track after 1, 2 and 3 s (m) and distance on ergometer after 1, 2 and 3 s (m)	19	High

STROBE, strengthening the reporting of observational studies in epidemiology; SCI, patients with spinal cord injury; WC, wheelchair; AB, able bodied persons; C, cervical level; T, thoracic level; L, lumbar level; IWBF class, International Wheelchair Basketball Federation class; CoP, centre of pressure; AIS, American Spinal Injury Association (ASIA) Impairment Scale [54].

Articles with a total STROBE score ≥15 were included in the analysis.

### Findings of the review

Twelve articles fulfilled the predetermined minimum of 15 reported items on the STROBE checklist (see Table 1) [29-40]. All 12 used a cross-sectional design. All but one study [40] was restricted to patients with SCI. And trunk impairment was defined by the America Spinal Injury Association (ASIA) score [41] in nine of the 12 studies [31,32,40].

In relation to activities determining proficiency in wheelchair court sports, the following three activities were described in the included articles: reach, maintaining balance after external perturbation and acceleration. No studies were found that assessed steady state propulsion, change of direction or tilting the chair.

Reach was assessed in a total of nine studies [29-34,37-39]: forward reach in five studies [33,34,37-39] and multidirectional reach in four studies [29-32]. Persons who were able bodied could reach further than those with SCI [33,34,37,38]. No distinction was found in forward reaching distance between persons with different levels of SCI as determined by the ASIA score (high thoracic T2-T8, low thoracic T9-T12 and lumbar L1-L5) [33,34,37]. Only one study reported further displacement of the centre of pressure (CoP) in persons with low thoracic SCI (T9-12) compared to persons with high thoracic SCI (T2-8) [38]. In multidirectional reach, differences in reaching in all directions summed in a single measure were found, with better reach in able bodied persons than in persons with SCI [32]. If a single measure for each direction was given, there was better reaching in almost all single directions in able-bodied persons compared to persons with SCI [31,32]. Also, there was better reach in persons low thoracic SCI than in persons with high SCI [30]. Similarly, persons with SCI with voluntary trunk muscle contractions could reach further than persons with SCI without trunk muscle contractions [32]. No difference was found in single direction measures between subgroups of persons with SCI for reaching in the lateral directions to both sides [29,31], to the left side [32] and for oblique directions to the left side [29,32].

Maintaining balance after external perturbation by tilting a platform on which the person was seated in a wheelchair, was described in two studies [35,36]. In tilting the platform in the sagittal or the frontal directions, those who were able bodied did not lose balance in any of the tilting accelerations (2 and 4 m/s^2^) or at any of the tilting angles. Persons with SCI all lost balance; the tilting angle in which balance loss occurred was higher in the low accelerations than in the high accelerations for all persons with SCI. Persons with paraplegia (T2-9) lost balance at a larger tilting angle in both directions than persons with tetraplegia (C5-7).

Only one included study explored acceleration in wheelchair racing [40]. This was the only study that included persons with other health conditions than SCI. More important, this was the only study that assessed the relation between impairment, irrespective of the health condition causing the impairment and activities. Trunk impairment was defined by a clinical test for trunk muscle strength. No difference in acceleration was found between athletes with full trunk muscle strength and those without full trunk muscle strength.

Four studies assessed the impact of equipment on performance in individuals with trunk impairment. Two studies assessed wheelchair set up, chair inclination [33] and the use of a rigid versus an elastic footrest [42] in relation to forward reach. No difference in reach was found for any of these adaptations to chair set up for either persons who were able bodied or those with SCI. One study examined the effect of thigh and chest belts on multidirectional reach [31]. In persons with SCI, reach increased in all directions by using a chest belt and to a lesser extent by using a thigh belt. Reach decreased by using a belt in persons who were able bodied. One study examined the use of functional electro stimulation (FES) and reported an increase in reach with FES application with both lifting a light and lifting a heavier object [40].

## Discussion

### Summary of evidence

In this systematic review, the authors synthesised the evidence available in the literature on the impact of trunk impairment on the execution of wheelchair activities that determine the performance in court sports. As expected, all identified studies were observational studies. All but three studies defined trunk impairment based on SCI lesion level and not on the biomechanical impairment types consisting of trunk muscle strength, coordination of the trunk and range of movement of the trunk. The evidence reported in the included studies indicates that persons who are able bodied can reach further forward than persons with SCI [33,34,37,38]. The difference in forward reaching distance between persons with different levels of SCI was conflicting, with no difference found in three studies [33,34,37] and larger reach found in one study in persons with low thoracic SCI compared to those with high thoracic SCI [38].

For multidirectional reach, if a sum measure for all directions was used, the following differences were found: (1) those who were able bodied showed better reach than persons with SCI. (2) Persons with SCI who could partially use their trunk muscles had better reach than those who had paralysed trunk muscles [31,32]. If separate directions were analysed, persons who were able bodied showed a larger reach than persons with SCI in almost all directions. For subgroups of persons with SCI, two studies found no differences in the left-right direction [29,31] and one did not find a difference in the left direction [32]. Two studies reported no differences in left oblique directions, but did find differences in the right oblique directions [29,32]. Consequently, oblique directions seem to be the most sensitive directions to discriminate in dynamic balance between persons with different levels of SCI. The difference between left and right oblique directions may be explained because reaching to the dominant right side is a more practised movement. Because of the limited number of participants per study, the power may have been too low to detect smaller differences to the non-dominant side. As a result, the authors suggest all reaching directions could possibly be affected by lesion level of SCI in optimally trained athletes.

There was also evidence to support that persons who are able bodied do not lose balance in perturbation with any force impact through acceleration in the lateral and forward direction in a seated position. The force impact that was applied (maximum acceleration of 4 m/s^2^) seems to match the impact of deceleration from maximum speed to stop in wheelchair court sports [12-14]. Persons with thoracic SCI do lose balance in perturbations in both the frontal and the lateral directions with this impact. Moreover, persons with cervical SCI lose balance with a lower impact than persons with thoracic SCI [35,36].

The only study exploring the relationship between trunk muscle strength and acceleration in wheelchair racing found no difference between athletes with full trunk muscle strength and athletes without full trunk muscle strength [40]. No literature was found for all other wheelchair activities of interest for this review, steady state propulsion, change of direction or tilting the chair.

The limited findings of this systematic review are difficult to generalise to athletes who compete in wheelchair court sport, because almost all study participants had complete SCI with trunk impairment defined according to the ASIA score [41]. The ASIA score is based on medical examination of strength and sensation. However, the trunk level (thoracic) is defined according to impairment in sensation only. The ASIA score only reflects impairment in trunk muscle strength in athletes with complete SCI in whom the level of impaired sensation equals the level of motor impairment. For impairment types other than trunk muscle strength (range and coordination), no information was found. The distribution of impairment in trunk muscle strength is typical for those with SCI, depending on lesion level [41]. However, impairment in trunk muscle strength can have a different distribution in individuals with other medical conditions, which may result in a different impact on activities. The authors of this systematic review could only report on evidence of effect of lesion level in SCI on reach and balance after external perturbation and not for any other activities that determine performance in wheelchair court sports.

A growing issue in the paralympic movement is the impact of equipment on performance. In this systematic review, equipment was defined as a covariate in relation to the study goal. Athletes are competitive by nature and will try to enhance their performance as well as limit the impact of impairment on performance by using optimised equipment. This use of equipment is sport specific and often based on experience. However, the equipment permitted in wheelchair court sports shows similarities across sports [12-14]. Studies in this review explored the use of belts and straps, wheelchair set up, seating configuration and functional electrical stimulation (FES). Two studies assessed the possible influence of equipment in relation to trunk impairment [36,38] though equipment that is not used for wheelchair court sports was tested [13,16]. The only study that assessed equipment used in court sports found that multidirectional reach improves in athletes with SCI by the use of a thigh or trunk belt [31]. Reach is only one of the many relevant activities for wheelchair court sport, and in addition to belting, the chair set up (position of the seat and the backrest and height of the backrest) is also believed to impact on reach [16]. In one study, FES of the trunk extensor muscles was used to improve unsupported seating and to lift objects with different weights [39]. Functional electrical stimulation primarily impacts on impairment in muscle strength. The use of implanted electrodes as a piece of equipment is new to moment in the paralympic movement. So far, there is no consensus if this equipment should be allowed during sport performance and whether or not this equipment should be part of athlete evaluation in classification [43]. On the other hand, in the study exploring the effect of trunk impairment on acceleration in wheelchair racing, athletes used their own custom designed wheelchair. It must be noted that a racing wheelchair seating configuration is quite different from the configuration of a wheelchair for court sports. Opposite to wheelchairs used for court sports, the racing wheelchair does not have rear castor wheels to prevent tipping when maximum force is applied on the wheel [44]. As a consequence of the absence of the castor wheel, the racing athletes in this study may not have been able to use maximum force in accelerating, because of the risk of tipping backward. This limits the generalisation of the results of this study regarding the impact of trunk impairment on acceleration to wheelchair court sports. Additional research is necessary to examine the acceleration in a court sports wheelchair with the typical rear castor wheels to determine if trunk muscle strength does impact on acceleration when tipping of the chair is prevented.

So far, the findings of this review provide limited support to the experience of wheelchair athletes and empirical information, supporting there is a significant impact of equipment on performance in wheelchair sport [18,45].

### Strengths and limitations

This systematic review has number of strengths. First, this study synthesised existing literature about the impact of trunk impairment on performance in wheelchair court sports using a strict set of inclusion and exclusion criteria. The methodological quality of all relevant studies was assessed using the STROBE checklist which is accepted as international standard for reporting of observational research [22]. The study was reported according to the MOOSE [21], which is accepted as a standard for meta-analysis. This strict approach enables replicating the study and extending it in the future if new evidence becomes available. Second, several types of potential bias were acknowledged and addressed in selecting studies for this review prior to the quality assessment, including publication bias, selection bias and quality assessment bias. The authors tried to minimise publication bias by manually searching abstracts of all conferences over the past 8 years that were relevant in relation to the objectives of this review. Only three potentially relevant abstracts were identified. Based on the lack of relevant unpublished information, it is unlikely that publication bias played a major role in this review. Furthermore, selection bias for study inclusion was addressed by independent literature searches in multiple databases completed individually by the two researchers using the same search terms. Bias in methodological quality assessment was minimized by the independent assessment of study quality by the two researchers (VA and AH). If there was no consensus between the two researchers, a consensus procedure with all authors was used for the final decision. Finally, bias in results and conclusions could have been introduced by the choice of method for quality assessment. By the authors’ knowledge, the STROBE guideline provides the only checklist available for methodological assessment of observational studies. The authors of the STROBE checklist state that “good reporting reveals the strengths and weaknesses of a study and facilitates sound interpretation and application of study results”. “The aim of the STROBE guideline is to ensure clear presentation of what was planned, done and found in observational studies” [23]. However, the STROBE recommendations do not provide a guideline or threshold for making decisions on inclusion of studies in a systematic review. In this systematic review, the authors chose a cut-off score for sufficient methodological quality for inclusion based on a study that determined the average score of present items in observational studies in high-quality medical journals, i.e., 69% [24].

There are several limitations to be considered when interpreting the results. All studies included small populations, which limited the study power. Possible effects of trunk impairment on the execution of wheelchair activities are not likely detected based on this limited power per study. Because the definition of trunk impairment and the outcome measures were different for each study, pooling of data to increase the power was not possible.

There are several aspects in the way tests were executed in the selected studies that may have caused bias in the results, including differences in measuring sitting balance, seating positions, use of belts and the experience level of the study participants. There were differences in the position of the arms during the measurement of sitting balance. One study allowed arm movements to compensate during trunk movements [29]. Most studies had a prescribed position of the arms, but they differed from a hand on the thighs position, which can support to some extent in forward movements [32], actively reaching with one [29] or both arms to a target [33,34,38,42], lifting a ball [31], or lifting objects with low and higher weight [39], to crossing the arms in front of the chest [35,36]. It is known that persons with a lack of trunk muscle strength can compensate with the recruitment of shoulder and arm muscles [37,46,47]; therefore, the difference in arm position can have caused bias in these results. Also, there were differences in seating position from the use of a flat surface with the knees and the hips in 90^°^ flexion with feet supported [29,30] to sitting in a standardised wheelchair [35,36]. Although no evidence for the impact of seating position was found in the present review, there is anecdotal evidence suggesting that seating position does impact on the execution of wheelchair activities [18]. Last but not least, contrary to most studies where persons perform without a belt, in two studies, persons were provided with a lap belt [35,36]. Because of the possible impact of different arm and seating positions and the use of a lap belt on reach and perturbation, the results should be interpreted with caution. For future research on trunk impairment in wheelchair activities, the positioning of the athlete, the positioning of the arms and the use of equipment should be standardised. Preferably for application to classification in sport, these positions and equipment should be relevant to the sport of which the activities are assessed.

Finally, the majority of studies included patients while only one study included trained athletes [40]. Experience in using a wheelchair is known to have a significant impact on the execution of wheelchair activities; consequently, studies including participants with very limited experience in manual wheelchair use were excluded [48]. Nevertheless, a significant difference in skills between patients from a hospital or rehabilitation centre and optimally trained athletes is well established [15]. Consequently, there are limitations in deducting the impact of trunk impairment on performance in athletes from the included studies, using patients or persons who had limited experience in wheelchair mobility. Additional research using athletes is required to generalise the results to wheelchair court sports. There are important obstacles being able to obtain appropriate samples in classification research in wheelchair court sports. These obstacles include the limited number of athletes, spread over large areas and limited financial resources for athlete training and research. Consequently, adequate populations of athletes cannot likely be tested or studied outside of major competitions. To overcome these obstacles, effort should be given to promote future testing around these competitions and to enable athlete participation in research.

## Conclusions

The literature provided limited information to aid in the development of evidence-based classification of trunk impairment in wheelchair court sports. As expected, additional research is needed, particularly for athletes with other health conditions than SCI resulting in impairment types other than trunk muscle strength. However, deficits in the current evidence were defined and can be used to guide future research in this field. The authors recommend the following for further research in wheelchair court sports: 1) Development of a test or scale for trunk impairment, comprising all biomechanical impairments of the trunk (trunk muscle strength, trunk coordination and trunk range of motion) [2]. This test needs to provide clear data for trunk impairment independent of the health condition causing the impairment. 2) Development of standardised tests for wheelchair activities that impact on wheelchair court sports performance. 3) Controlling for other factors that can impact on performance in wheelchair activities in sport, such as wheelchair configuration, positioning and strapping. 4) Facilitation of networking between researchers in several wheelchair sports needs to be facilitated to overcome limitations in resources and numbers of participants.

### Statement of ethics

The manuscript does not contain clinical studies or patient data.
